# P-1921. Nursing Home Resident Hospitalizations Related to Infection, Medicare Claims, 2022

**DOI:** 10.1093/ofid/ofaf695.2090

**Published:** 2026-01-11

**Authors:** Kelly M Hatfield, Lindsey Walker, Erika Wallender, Nicola Thompson, Cheri Grigg, Joseph D Lutgring, Sujan Reddy, Kara M Jacobs Slifka

**Affiliations:** Centers for Disease Control and Prevention, Atlanta, GA; Centers for Disease Control and Prevention; Oak Ridge Institute for Science and Education (ORISE), Oak Ridge, Tennessee, USA., Atlanta, Georgia; CDC, Atlanta, Georgia; CDC, Atlanta, Georgia; Division of Healthcare Quality Promotion, Centers for Disease Control and Prevention, Atlanta, Georgia; Division of Healthcare Quality Promotion, Centers for Disease Control and Prevention, Atlanta, Georgia; CDC, Atlanta, Georgia; Centers for Disease Control and Prevention, Atlanta, GA

## Abstract

**Background:**

Quantifying the relative burden of infections among hospitalizations from nursing home residents can inform prevention strategies.

**Figure:**
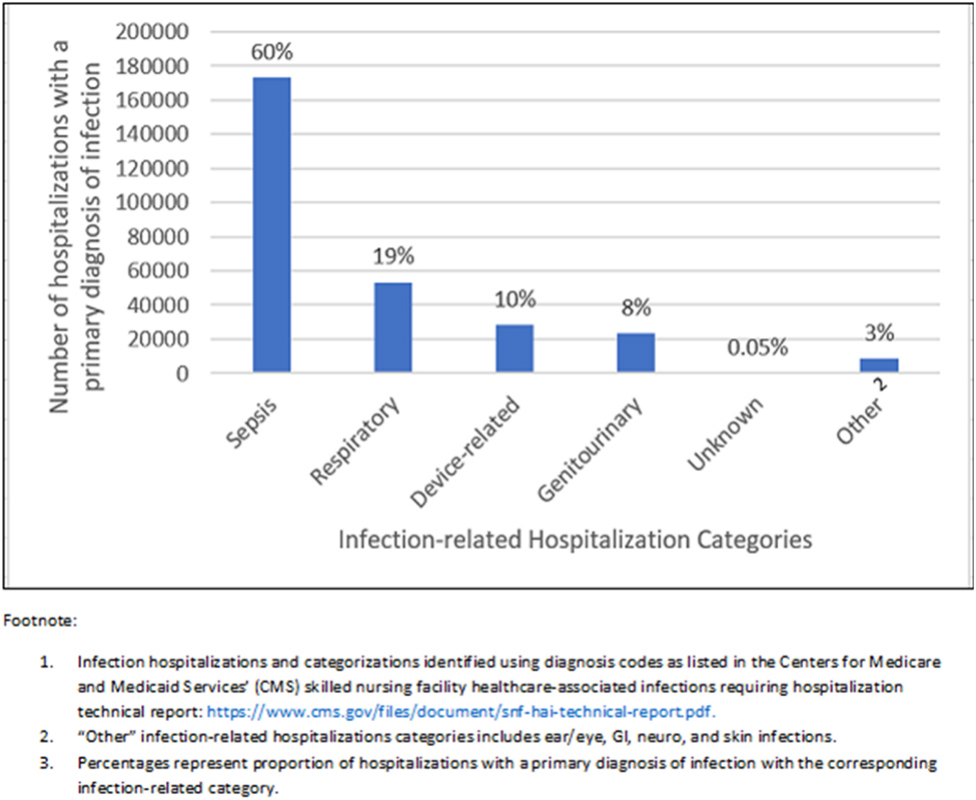
Infection hospitalizations stratified by primary diagnosis categorization using the diagnosis codes listed in Centers for Medicare and Medicaid Services’ skilled nursing facility healthcare-associated infections requiring hospitalization measure (1).

**Figure:**
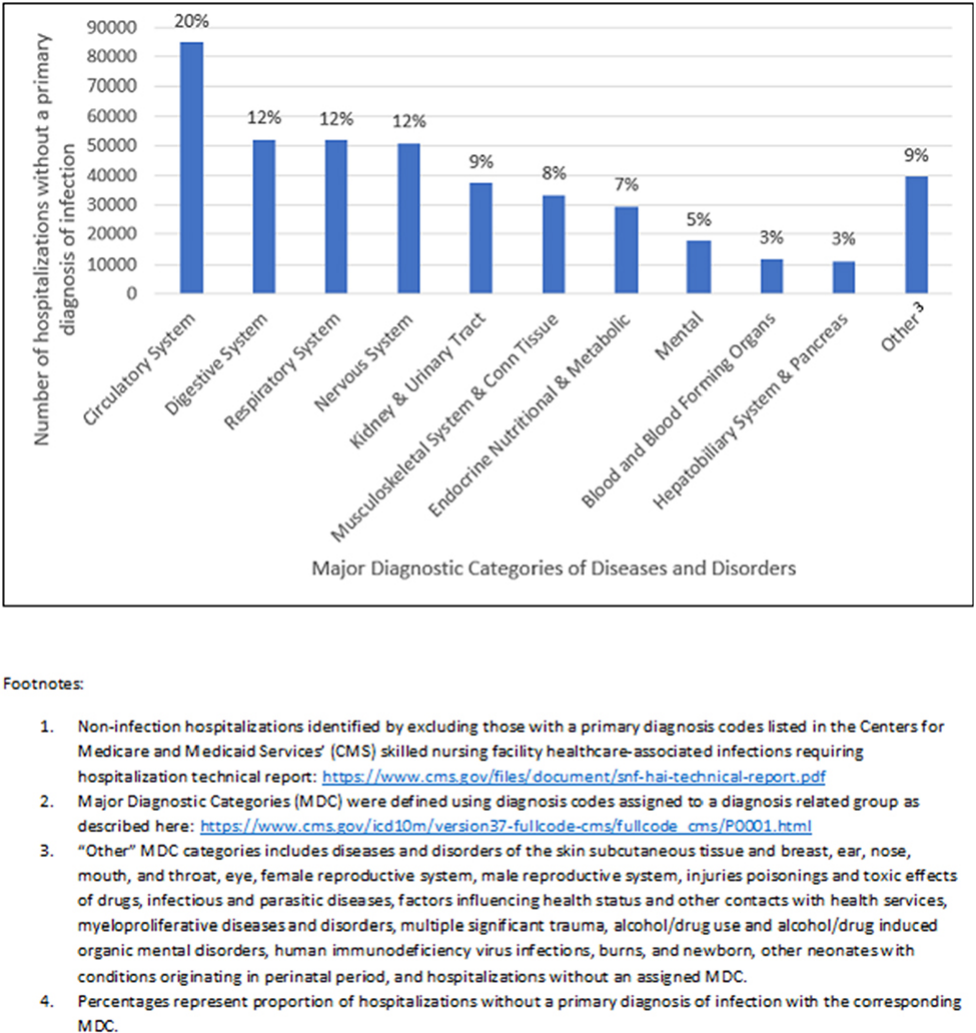
Characterization of hospitalizations without a primary diagnosis of infection (1) grouped by major diagnostic category (2).

**Methods:**

We used MedPAR files to identify Medicare claims for unplanned inpatient hospital admissions. We included all hospitalizations with an admission date during or within one day of a nursing home stay (identified using assessments in the Minimum Dataset 3.0). We categorized infection-related hospitalizations by type using the primary diagnosis code, similar to a Medicare skilled nursing facility quality measure, *healthcare-associated infections requiring hospitalization*. We described the remaining non-infection hospitalizations using Major Diagnostic Categories (MDCs). Resident age, sex, hospitalization costs, and other characteristics were compared among those with infection-related and non-infection hospitalizations using chi-square and Wilcoxon rank-sum tests.

**Results:**

We identified 706,875 hospital admissions among 494,611 nursing home residents in 2022. Among these, 286,446 (41%) had a primary diagnosis of an infection. The most frequent infection-related categorizations included sepsis (n=173,013, 60%), respiratory (n=53,288, 19%), device-related (n=28436, 10%), and genitourinary infections (n=23,089, 8%) (Figure 1). Of the non-infection hospitalizations, the most common MDCs were diseases and disorders of the circulatory (n=84,890, 20%), digestive (n=52,174, 12%), respiratory (n=52,021, 12%), and nervous systems (n=50,893, 12%) (Figure 2). For all hospital admissions, the mean beneficiary age was 77 years, 393,562 (56%) were females, and 301,460 (43%) had been hospitalized within the previous 30 days.

Hospitalizations with a primary diagnosis of an infection had a longer median length of stay (6 vs 5 days), higher median claim cost ($10,905 vs $8,051), a higher proportion with intensive care unit admission (24% vs 12%), and higher proportion of death at discharge (14% vs 6%) than non-infection hospitalizations (all p< 0.001).

**Conclusion:**

Infection-related primary diagnoses constituted over 40% of all hospitalizations among nursing home residents and have increased morbidity and mortality. Prevention of infections among nursing home residents across healthcare settings may improve wellbeing.

**Disclosures:**

All Authors: No reported disclosures

